# Outcomes and Complications of Pars Plana Vitrectomy for Tractional Retinal Detachment in People With Diabetes: A Systematic Review and Meta-analysis

**DOI:** 10.1001/jamaophthalmol.2022.5817

**Published:** 2023-01-12

**Authors:** Philip McCullough, Ajay Mohite, Gianni Virgili, Noemi Lois

**Affiliations:** 1 Wellcome-Wolfson Institute for Experimental Medicine, Queen’s University Belfast, Belfast, Northern Ireland, United Kingdom; 2 Department of Ophthalmology, The Belfast Health and Social Care Trust, Belfast, Northern Ireland, United Kingdom; 3 Centre for Public Health, Queen’s University Belfast, Belfast, Northern Ireland, United Kingdom; 4 Department of Neuroscience, Psychology, Drug Research and Child Health, University of Florence, Florence, Italy

## Abstract

**Importance:**

Tractional retinal detachment (TRD) occurs in approximately 5% of people with proliferative diabetic retinopathy and poses a threat to vision. Pars plana vitrectomy (PPV) is the treatment of choice for TRD.

**Objective:**

To determine anatomic and functional outcomes of PPV for the treatment of TRD in people with diabetes (dTRD).

**Data Sources:**

MEDLINE and Embase were searched systematically from January 1, 2000, to February 20, 2022. In addition, a reference list of eligible studies were screened.

**Study Selection:**

Eligible studies were those published in English, those reporting outcomes of PPV for dTRD, and those that included more than 25 eyes and with a minimum follow-up of 3 months.

**Data Extraction and Synthesis:**

Preferred Reporting Items for Systematic Reviews and Meta-analyses (PRISMA) guidelines for data extraction/synthesis were followed, and the National Institute for Health quality assessment tool was used to assess risk of bias. Study eligibility was determined independently by 2 reviewers; data extraction was conducted by 1 reviewer and entries checked for accuracy by another. Data were pooled using a random-effects model.

**Main Outcomes and Measures:**

Main outcomes included rate of failure of retinal reattachment following 1 surgery and final visual acuity (VA). The association of baseline patient characteristics and surgical maneuvers with postoperative surgical outcomes was investigated.

**Results:**

Of the 406 studies identified, 38 (3839 eyes) were eligible and included for analysis. Patients had a median (IQR) age of 52.2 (49.6-55.7) years. In the studies reporting patient sex (31 of 38 studies), 1441 were female individuals (50.1%). The overall failure rate of retinal reattachment after 1 surgery was 5.9% (95% CI, 1.4%-8.3%), and the mean final VA was 0.94 (95% CI, 0.82-1.05) logMAR (approximate Snellen equivalent, 6/53; 95% CI, 6/39-6/71). People with higher preoperative VA achieved higher postoperative vision (0.66 logMAR worse final vision; 95% CI, 0.39-0.84 per 1.0 logMAR worse at baseline; *P* <.001). On multivariable analysis, no other patient characteristics or surgical variables had a statistically significant association with outcomes.

**Conclusions and Relevance:**

Results of this systematic review and meta-analysis suggest that PPV was an effective strategy to achieve retinal reattachment in people with dTRD. Given that higher preoperative VA was the only factor associated with higher postoperative vision, early intervention should be considered and discussed in detail with patients. Overall, final postoperative VA remains low, and patients should be counseled on the guarded prognosis of dTRD.

## Key Points

**Question:**

What are the outcomes of pars plana vitrectomy for the treatment of diabetic tractional retinal detachment (dTRD), and what patient and surgical characteristics determine them?

**Findings:**

In this systematic review and meta-analysis of 36 studies (3720 eyes), retinal reattachment is high, but final vision is low. Higher baseline vision was associated with higher postoperative vision.

**Meaning:**

Study findings suggest that pars plana vitrectomy is associated with high anatomic reattachment but limited final vision postoperatively, which may be useful for the counseling of patients with dTRD; given that higher preoperative visual acuity is associated with higher postoperative vision, early intervention should be considered and discussed with patients.

## Teaser

This systematic review and meta-analysis investigates the outcomes of pars plana vitrectomy for the treatment of diabetic tractional retinal detachment.

## Introduction

Diabetic retinopathy (DR) affects approximately 93 million people globally; one-quarter of those experience vision loss.^1^ Tractional retinal detachment (TRD) is the most serious complication of DR. TRD results from contraction of fibrovascular membranes, which are adherent to the retina. In the hyperglycemic environment, fibrovascular membranes are formed in response to the secretion of angiogenic factors, including vascular endothelial growth factor (VEGF).^2^

Despite the introduction of diabetic eye screening programs, improvements in diabetic control,^3^ and available treatments for proliferative DR (PDR), many people living with diabetes still develop severe complications, including TRD. Approximately 5% of patients with PDR require pars plana vitrectomy (PPV) for TRD despite having been treated with panretinal photocoagulation.^4^

PPV for TRD involves the removal of vitreous and fibrovascular membranes, relieving tractional forces on the retina. In the last 25 years, there have been advances in surgical techniques, including small-gauge vitrectomy^5^ and new tamponade agents^6^; anti-VEGF medications are also used preoperatively and intraoperatively.^7^ Patient demographics have changed over time.^7^ Furthermore, improvements in metabolic control with the use of new oral hypoglycemic agents and glucose monitoring devices have occurred.^8^ All of these issues have the potential to influence surgical outcomes of TRD in people with diabetes.

The purpose of this study was to evaluate anatomic and functional outcomes and complications of dTRD repair by undertaking a systematic review and meta-analysis of the existing literature. In addition, we investigated whether baseline patient characteristics and surgical maneuvers were associated with these outcomes.

## Methods

### Sources and Search Methods

The protocol for this meta-analysis was developed following the Preferred Reporting Items for Systematic Reviews and Meta-analyses (PRISMA) reporting guidelines^9^ and uploaded in the International Prospective Register of Systematic Reviews (PROSPERO).^10^ A search of MEDLINE and Embase databases was conducted for potentially eligible studies using a predefined search strategy (eFigure 1 in Supplement 1). The final search took place on February 20, 2022. Reference lists of eligible studies were also scrutinized for potentially eligible studies.

### Eligibility Criteria

Studies were eligible for inclusion if they were randomized clinical trials (RCTs), case-control studies, and prospective or retrospective before and after studies presenting outcomes of PPV for dTRD published from January 1, 2000 (to ensure capturing all studies using small bore vitrectomy), to February 20, 2022 (last search done), and followed up patients for a minimum of 3 months. Due to a limited time frame and funding for this study, only articles published in English were included; planning (at the time of the study conception) translations of articles from any language (necessary to maintain equity) that we could have encountered to English did not seem feasible and, thus, was not undertaken. Studies presenting outcomes of PPV for nondiabetic TRDs or other vitreoretinal disorders; those evaluating PPV for other diabetic related complications (eg, vitreous hemorrhage) without concomitant TRD; studies presenting outcomes after repeated vitrectomy (rather than the primary surgery); and case reports and case series including less than 25 eyes were excluded.

### Search Strategy

Search results were independently reviewed by 2 of 3 reviewers (P.M., A.M., N.L.). Based on titles and abstracts, studies were classified as potentially eligible or ineligible. Full-text articles of potentially eligible studies were obtained and classified as eligible or ineligible by 2 of 3 independent reviewers (P.M., A.M., N.L.). Reference lists contained in manuscripts presenting eligible studies were also scrutinized for potentially eligible studies; if found, these were subject to the same process. At all stages, discrepancies were solved by discussion or with the intervention of an arbitrator.

### Data Extraction

Data were extracted by 1 reviewer (P.M.) and checked by a second reviewer (A.M.) for accuracy. The following data, if available, were extracted at 3 months (±1 month) and 12 months (±3 months) postoperatively (other time points were considered if available): number (percentage) of people (eyes) with a flat retina after a single surgery and number (percentage) of people (eyes) with a flat retina after more than 1 surgery; best-corrected visual acuity (BCVA); proportion of people (eyes) achieving VA of 0.30 logMAR (approximate Snellen equivalent, 6/12) or better and 1.0 logMAR (approximate Snellen equivalent, 6/60) or worse; and intraoperative and postoperative complications. Information regarding age and sex was retrieved from eligible studies. However, data regarding other demographic characteristics (eg, race and ethnicity) were not extracted as these were only infrequently reported. When available, data pertaining to preoperative patient presenting characteristics, surgical maneuvers used during PPV, use of preoperative or intraoperative anti-VEGF injections, and number of surgeries required for retinal reattachment were also retrieved. If required, study authors were contacted for data clarification.

### Risk of Bias and Quality Assessment

Risk of bias was evaluated using the National Institutes for Health quality assessment tools applicable to the relevant study design.^11^ Risk of bias was assessed by 2 independent reviewers (P.M., A.M.). Disagreements were resolved via discussion or with the intervention of an arbitrator. The National Institutes for Health quality assessment tools rate studies as follows: (1) “good” denoting low risk of bias, “fair” denoting some risk of bias but not enough to invalidate the study, and “poor” denoting significant risk of bias.

### Statistical Analysis

We fitted meta-analyses of proportions using the metaprop_one command in Stata, version 16.1 (StataCorp) with specifications according to Schwarzer et al.^12^ We fitted a random-intercept logistic-regression model. In addition, we used a maximum-likelihood estimator for *τ*^2^, a logit transformation of proportions, and a Clopper-Pearson CI for individual studies. Continuous variables (eg, mean final VA) were pooled using an inverse variance method with restricted maximum likelihood estimation of random effects.^13^

Between-study heterogeneity was assessed graphically, inspecting the overlap of study CIs and *I*^2^ values. Preplanned heterogeneity investigation was conducted adding study-level categorical covariates to the model. Continuous study-level variables (eg, mean age) were dichotomized using median values and used as covariates in the model. Due to the distribution of data, the use of preoperative or intraoperative anti-VEGF medications was dichotomized as studies with 100% usage and those with less than 100% usage. Gauge was dichotomized as studies using exclusively standard 20-gauge vitrectomy and those using exclusively small-gauge vitrectomy (23, 25, and 27 gauge). To allow a comparison of outcomes from studies published within the last 5 years with older studies, a post hoc analysis was undertaken dichotomizing studies as published during or after 2016 and those published before 2016. When using data presented in RCTs, all study arms were included if pertinent to this review. Simple descriptive statistics were used to present postoperative complications.

## Results

### Study Selection

In total, 406 studies, of which 92 were duplicates, were identified via database searches and reference lists of included studies (eFigure 2 in Supplement 1). After initial screening, 98 potentially eligible studies were identified. Two, both published in the European Vitreoretinal Society (EVRS) Educational Electronic Journal, where irretrievable from Queen’s University library (Belfast, UK) and The British Library (London, UK)^14,15^ and could not be assessed. After full-text evaluation of all other studies, 38 (with 3839 eyes) were found eligible and included.^5,6,16,17,18,19,20,21,22,23,24,25,26,27,28,29,30,31,32,33,34,35,36,37,38,39,40,41,42,43,44,45,46,47,48,49,50,51^ Patients had a median (IQR) age of 52.2 (49.6-55.7) years. In the studies reporting patient sex (31 of 38 studies), 1441 were female individuals (50.1%), and 1437 (49.9%) were male individuals. A summary of these 38 studies and of baseline characteristics of patients included can be found in eTable 1 and eTable 2 in Supplement 1. A summary of the outcomes evaluated in this review reported in eligible RCTs and prospective studies only (ie, the robust study designs) is presented in eTable 3 in Supplement 1. For RCTs, as all arms presented were pertinent to this review, data from all arms were extracted and analyzed. Surgical maneuvers used in each included study are summarized in eTable 4 in Supplement 1.

### Outcomes of TRD Repair

**Figure 1.**
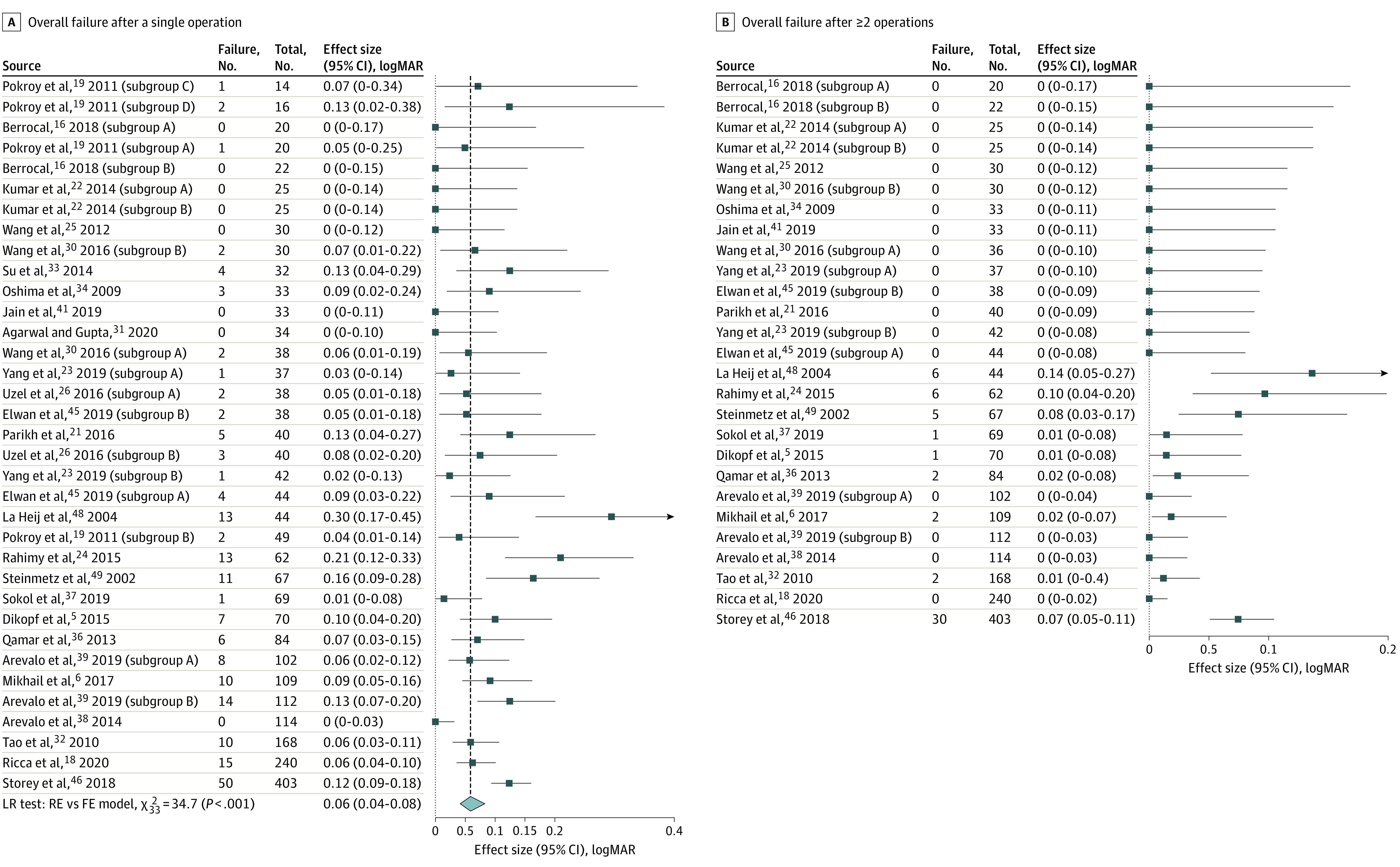
Meta-analysis of the Proportion of Eyes With Overall Failure, Sorted by Sample Size A, Meta-analysis of the proportion of eyes with overall failure after a single surgery and after 2 or more surgeries (B). Some studies included in the meta-analysis presented different groups based on the treatment performed. We have provided the results for these different groups separately as denoted by letters (ie, A, B, C…). A description of each group can be found eTable 1 in Supplement 1.

The meta-analysis of the overall rate of failure to obtain retinal reattachment after a single surgery included 25 studies^5,6,16,18,19,21,22,23,24,25,26,30,31,32,33,34,36,37,38,39,41,45,46,48,49^ (2344 eyes) (Figure 1A); the pooled estimate of failure was 5.9% (95% CI, 4.1%-8.3%). The meta-analysis of overall rate of failure to obtain retinal reattachment after more than 1 surgery included 21 studies^5,6,16,18,21,22,23,24,25,30,32,34,36,37,38,39,41,45,46,48,49^ (1564 eyes) (Figure 1B); the rate of failure was 0.7% (95% CI, 0.2%-2.3%).

**Figure 2.**
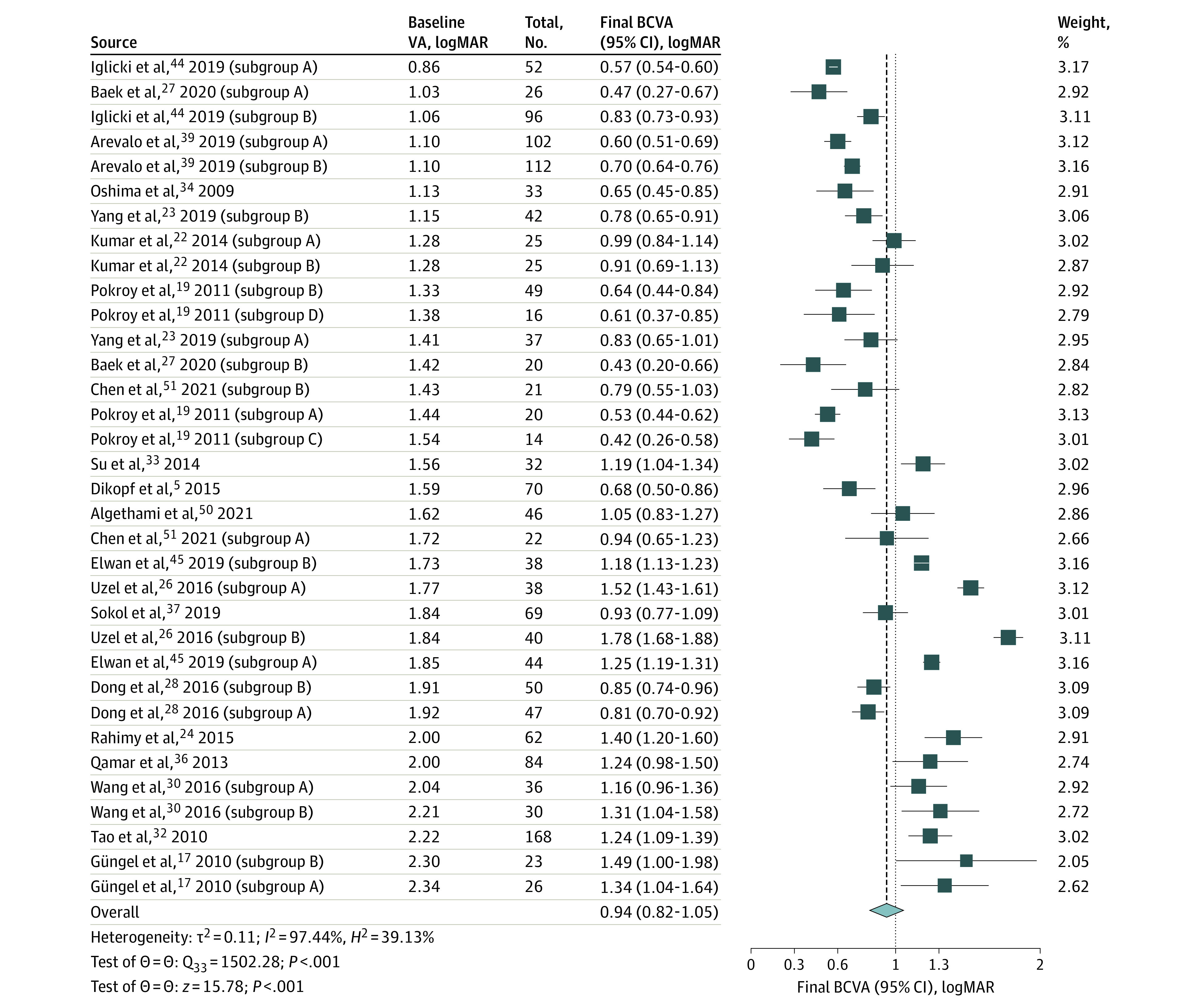
Meta-analysis of the Final Visual Acuity, Sorted by Baseline Vision Some studies included in the meta-analysis presented different groups based on the treatment performed. We have provided the results for these different groups separately as denoted by letters (ie, A, B, C…). A description of each group can be found eTable 1 in Supplement 1.

The meta-analysis of overall final VA included 20 studies^5,17,19,22,23,24,26,27,28,30,32,33,34,36,37,39,44,45,50,51^ (1526 eyes). The pooled final BCVA was 0.94 (95% CI, 0.82-1.05) logMAR (approximate Snellen equivalent, 6/53; 95% CI, 6/39-6/71) (Figure 2).

Other functional outcomes, including VA at 3 and 12 months postoperatively and number and proportion of eyes achieving VA of 0.30 logMAR (approximate Snellen equivalent, 6/12) or better and 1.00 logMAR (approximate Snellen equivalent, 6/60) or worse were considered but, due to a lack of data, meta-analysis was not possible.

### Influence of Baseline Characteristics and Surgical Maneuvers on Postoperative Outcomes

#### Anatomic Outcomes

On univariable analysis, 4 of 14 covariates investigated were statistically significantly associated with risk of failure of retinal reattachment with a single surgery (Table 1), including vitreous hemorrhage, lens status at presentation, use of preoperative or intraoperative anti-VEGF medications, and instrumentation gauge. Preoperative vitreous hemorrhage and lens status (phakic) increased the risk of failure (vitreous hemorrhage: median, 29.2%; IQR, 0-57.5%; *P* = .009 and phakic lens status: median, 87.7%; IQR, 71.6%-93.7%; *P* = .04), whereas use of preoperative or intraoperative anti-VEGF medications (failure rates in studies [n = 34] with 100% preoperative or intraoperative anti-VEGF use [7.7%; 95% CI, 5.3%-11.1%] vs those studies [n = 15] with <100% usage [3.3%; 95% CI, 1.5%-6.9%]; *P* = .02), and use of small gauge instrumentation (23, 25, and 27 gauge) (failure rate for studies [n = 14] using 20 gauge [8.7%; 95% CI, 5.3%-14.0%] vs those [n = 29] using 23, 25, or 27 gauge [3.6%; 95% CI, 1.9%-6.5%]) reduced this risk.

**Table 1.** Results of Univariable Meta-analysis Evaluating the Association of Baseline Patient Characteristics and Surgical Approaches With Failure to Achieve Retinal Reattachment After a Single Surgery

Covariate	Covariate, median (IQR)	Failure rate of studies below the covariate median		Failure rate of studies above the covariate median		*P* value for subgroup difference
		Study arms, No.	% (95% CI)	Study arms, No.	% (95% CI)	
Baseline patient characteristics						
Age, y	52.2 (49.6-55.7)	24	6.7 (4.3-10.3)	24	6.2 (4.2-9.2)	.80
Diabetes duration, y	14.9 (12.4-16.4)	10	6.6 (5.0-8.6)	10	7.1 (5.0-10.0)	.52
HbA_1c_, %	8.5 (8.2-8.9)	9	5.6 (2.0-14.6)	12	6.2 (3.5-10.8)	.84
Hypertension, %	73.0 (42.1-83.0)	8	5.2 (2.4-11.1)	8	7.7 (5.0-11.7)	.35
VH, %	29.2 (0-57.5)	24	3.9 (1.9-7.9)	25	9.1 (6.7-12.1)	.009
Attached macula, %	16.0 (0-42.2)	17	5.0 (2.4-10.2)	17	7.9 (5.7-10.8)	.20
Phakic, %	87.7 (71.6-93.7)	15	5.8 (3.9-8.6)	15	10.4 (7.3-14.5)	.04
Baseline VA logMAR	1.59 (1.28-1.84)	22	6.2 (4.3-9.0)	23	7.6 (5.1-12.0)	.44
Surgical maneuvers						
Silicone tamponade, %	25.3 (7.1-70.0)	25	5.4 (3.2-9.0)	25	7.3 (4.6-11.5)	.39
SF6/C3F8 tamponade, %	25.0 (0.0-55.0)	21	5.3 (2.6-10.6)	21	7.1 (4.6-110)	.53
None, BSS/air	6.0 (0.0-42.0)	20	5.0 (2.9-8.3)	21	8.2 (5.2-12.5)	.16
Preoperative/intraoperative anti-VEGF agent use (<100% vs 100%)						
Below 100%^a^	NA	34	7.7 (5.3-11.1)	NA	NA	.02
100%	NA	NA	NA	15	3.3 (1.5-6.9)	
Gauge (20 vs 23-27)						
20	NA	14	8.7 (5.3-14.0)	NA	NA	.03
23-27	NA	NA	NA	29	3.6 (1.9-6.5)	
Year of publication (2016+ vs <2016)						
Before 2016	NA	6.4 (3.4-11.4)	NA	NA	NA	.84
2016 or after	NA	NA	NA	5.9 (4.0-8.4)	NA	

Abbreviations: BSS, balanced salt solution; C3F8, octafluoropropane; HbA_1c_, hemoglobin A_1c_; SF6, sulfur hexafluoride; VA, visual acuity; VEGF, vascular endothelial growth factor; VH, vitreous hemorrhage.

^a^ A total of 25 study arms never used anti-VEGF agents preoperatively and intraoperatively, and 9 study arms used anti-VEGF agents in a median of 19% of patients.

The outcome of these variables was then investigated further using multivariable meta-analysis but found not to be statistically significant (eTable 5 in Supplement 1). Due to insufficient data, the association of baseline patient characteristics and surgical maneuvers with the risk of failure to achieve retinal reattachment after more than 1 surgery could not be investigated.

#### Functional Outcomes

**Figure 3.**
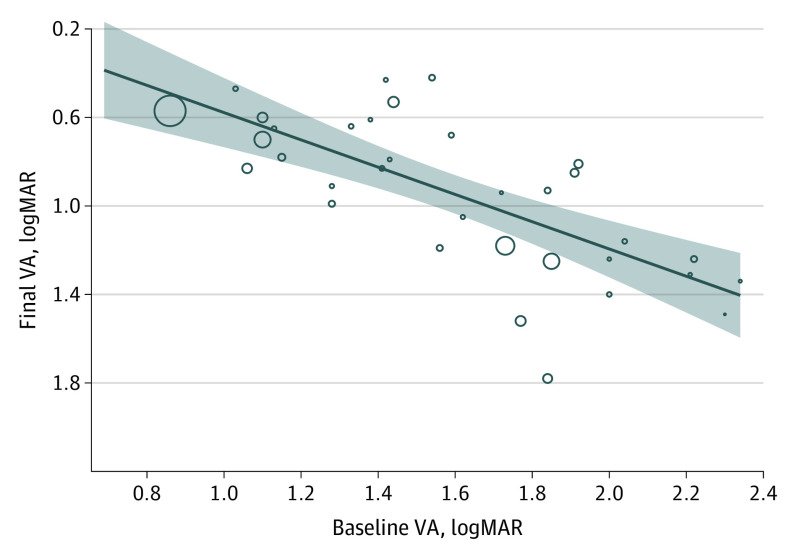
Graphical Presentation of Meta-Regression Results of Final Visual Acuity Based on Baseline Preoperative Visual Acuity Bubbles are proportional to study size, the dashed line is the prediction line, and the shaded areas are equal to 95% CI width.

On univariable analysis, 2 of 14 covariates investigated were found to be statistically significantly associated with final vision (Table 2), including hypertension, which appeared to have a protective association with borderline significance (median, 73.0%; IQR, 42.1%-83.0%; *P* = .04) and presenting vision, where eyes with higher preoperative VA achieved a higher final VA postoperatively (0.62 logMAR worse final vision; 95% CI, 0.39-0.84 per 1.0 logMAR worse VA at baseline; *P* < .001) (Figure 3). Only baseline VA was significantly associated with final VA (0.66 logMAR worse final vision; 95% CI, 0.41-0.93 per 1.0 logMAR worse VA at baseline; *P* < .001) in a multivariable model, which also included hypertension (0.03 logMAR; 95% CI, −0.19 to 0.25 logMAR; *P* = .81).

**Table 2.** Results of Univariable Meta-analysis Evaluating the Association of Baseline Patient Characteristics and Surgical Approaches With Final Vision

Covariate	Covariate median (IQR)	Final visual acuity of studies below the covariate median		Final visual acuity of studies above the covariate median		*P* value for subgroup difference
		Study arms, No.	LogMAR (95% CI)	Study arms, No.	LogMAR (95% CI)	
Baseline patient characteristics						
Age, y	52.2 (49.6-55.7)	24	0.97 (0.83-1.11)	24	0.96 (0.76-1.17)	.97
Diabetes duration, y	14.9 (12.4-16.4)	10	0.95 (0.67-1.23)	10	0.88 (0.57-1.20)	.75
HbA_1c_, %	8.5 (8.2-8.9)	9	0.83 (0.58-1.07)	12	0.64 (0.57-0.73)	.16
Hypertension, %	73.0 (42.1-83.0)	8	1.13 (0.64-1.62)	8	0.62 (0.53-0.70)	.04
VH, %	29.2 (0-57.5)	24	1.08 (0.82-1.35)	25	0.95 (0.78-1.12)	.42
Attached macula, %	16.0 (0-42.2)	17	0.88 (0.63-1.13)	17	1.11 (0.88-1.33)	.20
Phakic, %	87.7 (71.6-93.7)	15	0.97 (0.77-1.18)	15	0.94 (0.74-1.13)	.79
Baseline VA logMAR	1.59 (1.28-1.84)	22	0.70 (0.59-0.80)	23	1.20 (1.05-1.36)	<.001
Surgical maneuvers						
Silicone tamponade, %	25.3 (7.1-70.0)	25	0.82 (0.68-0.96)	25	1.03 (0.83-1.20)	.06
SF6/C3F8 tamponade, %	25.0 (0.0-55.0)	21	1.03 (0.82-1.23)	21	0.93 (0.76-1.10)	.51
None, BSS/air	6.0 (0.0-42.0)	20	0.95 (0.80-1.09)	21	0.98 (0.84-1.37)	.59
Preoperative/intraoperative anti-VEGF agent use (<100% vs 100%)						
Below 100%^a^	NA	34	1.00 (0.83-1.17)	NA	NA	.52
100%	NA	NA	NA	15	0.91 (0.72-1.11)	
Gauge (20 vs 23-27)						
20	NA	14	0.87 (0.63-1.11)	NA	NA	.19
23-27	NA	NA	NA	29	1.08 (0.89-1.22)	
Year of publication (2016+ vs <2016)						
Before 2016	NA	22	0.89 (0.58-1.19)	NA	NA	.23
2016 or after	NA	NA	NA	33	0.99 (0.79-1.20)	

Abbreviations: BSS, balanced salt solution; C3F8, octafluoropropane; HbA_1c_, hemoglobin A_1c_; SF6, sulfur hexafluoride; VA, visual acuity; VEGF, vascular endothelial growth factor; VH, vitreous hemorrhage.

^a^ A total of 25 study arms never used anti-VEGF agents preoperatively and intraoperatively, and 9 study arms used anti-VEGF agents in a median of 19% of patients.

The association of baseline patient characteristics and surgical maneuvers with other functional outcomes (VA at 3 and 12 months, number and proportion of eyes achieving VA of 0.30 logMAR [approximate Snellen equivalent, 6/12] or better and 1.00 logMAR [approximate Snellen equivalent, 6/60] or worse) was considered but, due to insufficient data, meta-analysis was not possible.

### Postoperative Complications

Across all included studies, 24 different postoperative complications were reported (eTable 6A-E in Supplement 1). The median number of postoperative complications reported per study was 2 (range, 0-11). Six studies (16%)^19,21,23,29,34,44^ did not report (ie, did not mention) postoperative complications, therefore, it was unclear if they occurred and, if so, at what rate.

Vitreous hemorrhage was reported in 22 studies (58%)^5,6,16,17,19,24,25,26,27,29,30,34,35,37,38,39,41,43,45,46,48,49^ (n = 1889 eyes) and occurred in 425 eyes (22.5%).

Only 1 study^21^ presented postoperative complications associated with the tamponade agent. In this study, silicone oil migration to the anterior chamber occurred in 4 eyes (10%) and under the conjunctiva and subretinally, each, in 2 eyes (5%). Emulsification of silicone oil occurred in 3 eyes (7.5%).

The most commonly reported postoperative complication was raised intraocular pressure, which was reported in 9 studies (24%)^5,6,27,29,32,36,40,44,45^ (n = 883 eyes) at a frequency of 10.5% (n = 93 eyes). Neovascular glaucoma was the most frequently reported postoperative complication associated with new vessel development and progression. It was reported in 14 studies (37%).^6,19,23,24,26,29,30,34,37,39,45,46,48,49^ (n = 1445 eyes), occurring in 76 eyes (5.3%). Endophthalmitis was reported in 7 studies (21%)^5,6,17,26,30,45,46^ but occurred in no eyes included in these 7 studies (n = 857 eyes).

Due to heterogeneity in the reporting of complications, meta-analysis was not possible. For the same reason, it was not possible to study the association of baseline patient characteristics/surgical maneuvers with the rate of postoperative complications. It was also not possible to assess cataract as a postoperative complication due to the heterogeneity in how it was reported in studies. In many studies, phacoemulsification or lensectomy was performed. However, it was not specified if the cataract was present preoperatively or whether it represented a surgical complication.

### Quality Assessment of Included Studies

Three RCTs were assessed using the RCT specific tool.^11^ Two where rated fair,^22,39^ and 1 was rated poor.^29^ The latter was deemed poor as it did not specify the method of randomization, whether there was allocation concealment, and did not have justification of the sample size. There was 1 case-control study^27^ and 1 observational cohort study. Both were assessed as being of good quality.

The remaining 35 studies were assessed using the before-after study tool^11^ of which 19 were graded good^5,16,17,24,26,27,30,32,33,34,36,37,40,44,46,48,49,50,51^; 15 fair^6,18,19,20,21,23,25,28,31,38,41,42,43,45,47^; and the remaining study as poor.^35^ This latter study was given a poor rating as outcomes and inclusion-exclusion criteria were not predefined. Additionally, statistical methodology was not reported, and it could not be determined owing to the masking of outcome assessors.

## Discussion

Results of this systematic review and meta-analysis suggest a high primary (with a single surgery) retinal reattachment rate (94%) following PPV for the treatment of dTRD, with retinal reattachment in 99% of eyes with repeated surgery. Despite this anatomic success, functional outcomes remained guarded, with final VA of 0.94 logMAR (approximate Snellen equivalent, 20/175), close to the level considered severe visual impairment (logMAR 1.0; Snellen equivalent, 20/200).^52^

PPV is used to treat other retinal disorders, including rhegmatogenous retinal detachment (RRD).^53^ A recent systematic review and meta-analysis^54^ of outcomes of PPV for RRD found a primary retinal reattachment rate of 72%, which is much lower than that for dTRD repair reported herein. Anatomic success after more than 1 surgery, however, was comparable (96%). Pooled meta-analysis of visual outcomes was not conducted in the RRD meta-analysis,^54^ but a postoperative VA of between 0.01 to 1.06 logMAR (approximate Snellen equivalent, 6/6 to 6/70) was reported. In comparison, postoperative vision following dTRD repair range from 0.86 to 2.34 logMAR (approximate Snellen equivalent, 6/44 to hand motions), which suggests that functional outcomes for dTRD were poorer than those for RRD repair. The RRD repair meta-analysis included exclusively RCTs and had no time or language restrictions and, thus, likely reflects more accurately anatomic and visual outcomes due to less chance of publication bias. The low postoperative vision achieved after PPV for dTRD could be explained by preestablished retinal damage, possibly the result of macular ischemia and/or neurodegeneration, in addition to permanent structural abnormalities that may take place as a result of the TRD itself. This is supported by the finding that eyes with higher vision preoperatively also achieved higher vision postoperatively. Retinal ischemia has been hypothesized by some authors as the reason for poor functional outcomes after dTRD repair.^20,21^ However, published studies did not evaluate in detail the causes of poor vision and, thus, these remained unclear and require further investigation. Given that higher presenting vision was the only factor associated with higher vision postoperatively, early intervention, before vision has been lost, should be considered and discussed with patients.

On univariable meta-analysis, less vitreous hemorrhage, the use of anti-VEGF agents, and smaller instrumentation gauge were associated with better anatomic outcomes. On multivariable analysis, however, no statistical significance was reached. This may be due to the high heterogeneity of patients with dTRD. Detailed phenotyping of patients should be undertaken and described in future studies as this would help interpreting results and improving the quality of future meta-analysis.

On univariable meta-analysis, we did not find anti-VEGF medications to be associated with final VA. A Cochrane systematic review and meta-analysis of RCTs and nonrandomized clinical trials, including trials evaluating anti-VEGF agents for the treatment of postoperative vitreous cavity hemorrhage, was unable to provide estimates of the association of anti-VEGF treatments with postoperative vision due to the heterogeneity of studies included in the review.^7^ However, a more recent systematic review and meta-analysis of RCTs showed an association between anti-VEGF medication use and improved visual outcomes after surgery.^55^ Furthermore, adjuvant anti-VEGF agents were shown to be associated with reducing intraoperative and postoperative early recurrent vitreous hemorrhage.^7,55^

Our review failed to show an association of gauge size with anatomic outcomes for dTRD. It is possible that, as just speculated, the heterogeneity of patients included in each of the eligible studies may have precluded detecting differences that may still exist. Small-bore vitrectomy has been widely adopted for vitreoretinal surgery and may have advantages, including reducing perioperative pain and inflammation.^22,56^

On full-text review of all identified studies classified initially as potentially eligible, 19 studies were excluded as authors had combined results for TRD repair with other indications for PPV, such as nonclearing vitreous hemorrhage. In the future, it would be important that outcomes of PPV are presented separately by indication as the prognosis of an eye with a nonclearing vitreous hemorrhage would be expected to be very different than that of an eye with a dTRD.

There was a high degree of variability and heterogeneity in the reporting of postoperative complications among included studies, making it challenging to assess the true complication rates associated with dTRD repair. Currently, there is no standardized tool for the reporting of intraoperative and postoperative complications specifically from dTRD. Recently, a new classification of complications of RRD surgery (Complications of Retinal Detachment Surgery [CORDS] severity classification) was developed using the Delphi consensus method, to aid classifying and quantifying severity of complications of RRD repair.^57^ Elaboration of a similar tool for dTRD would be advantageous to better understand harms related to its surgical repair and allow for comparisons of different surgical approaches for this condition.

### Limitations

This study has several limitations. Meta-analysis was undertaken with study data rather than individual participant data. It was, therefore, difficult to infer the association of baseline patient characteristics/surgical maneuvers with anatomic and functional outcomes. The number of eyes included was sizable, but the review was based on the evidence in the literature, which relies mostly on retrospective before and after studies. Although most studies were deemed to have low risk of bias, it is still possible that series achieving best surgical outcomes would be prepared and published, whereas less favorable results may not ever get written. We meta-analyzed all studies independently of their study design and did not undertake sensitivity analyses to determine whether results would have remained the same if only data from more robust study designs were to be considered. Furthermore, several studies used eyes, rather than patients, as the unit of analysis and did not adjust for within-participant correlation. This may have slightly inflated the precision of the estimates, but to a limited extent, because the number of patients in whom both eyes were included in studies was small (less than 20%). Although the results of this review may be helpful for the counseling of patients with dTRD before surgery, these limitations should be considered. Visual outcomes remain guarded even after successful retinal reattachment. If the poor vision is indeed the result of the intrinsic retinal damage from DR, further innovation in PPV techniques would unlikely improve functional outcomes. Earlier intervention may be helpful, but the focus should be on identifying people at high risk of developing dTRD and its prevention.

## Conclusions

Results of this systematic review and meta-analysis suggest that PPV was an effective strategy to achieve retinal reattachment in people with dTRD. Given that higher preoperative VA was the only factor associated with higher postoperative vision, early intervention should be considered and discussed in detail with patients. Overall, final postoperative VA remains low, and patients should be counseled on the guarded prognosis of dTRD. Although earlier surgery may be helpful, there should be a focus on identifying patients at high risk of developing dTRD and its prevention.

## Notes

Supplement 1. **eFigure 1.** Search Structure **eFigure 2.** PRISMA Flow Diagram **eTable 1.** Summary of Study Details, Sorted by Study Design **eTable 2.** Summary of Baseline Patient Characteristics of Included Studies, Sorted by Study Design **eTable 3.** Summary of Main Outcomes Evaluated in This Review Reported in Eligible RCTs and Prospective Studies (n=8) **eTable 4.** Summary of Surgical Details of Included Studies, Sorted by Study Design **eTable 5.** Results of Multivariable Meta-analysis Evaluating the Effect of Baseline Patient Characteristics and Surgical Approaches on Failure to Achieve Retinal Reattachment Following 1 Surgery **eTable 6.** Frequency of Postoperative Complications

Supplement 2. Data Sharing Statement
