# Supplementary material for: Outcomes and Complications of Pars Plana Vitrectomy for Tractional Retinal Detachment in People With Diabetes: A Systematic Review and Meta-analysis
Source: JAMA Ophthalmol. 2023 Jan 12;141(2):186–95. doi: 10.1001/jamaophthalmol.2022.5817 (PMC9857853; doi:10.1001/jamaophthalmol.2022.5817)
Supplement: Supplement 2. — Data Sharing Statement [file jamaophthalmol-e225817-s002.pdf]

McCullough P, Mohite A, Virgili G, Lois N. Outcomes and complicaitons of pars plana vitrectomy for tractional retinal detachment in people with diabetes: a systematic review and meta-analysis. *JAMA Ophthalmol.* Published online January 12, 2023. doi:10.1001/jamaophthalmol.2022.5817

Data Sharing Statement

Data

Data available: No

Additional Information

Explanation for why data not available: This is an aggregate meta-analysis of published studies
