# Supplementary material for: Outcomes and Complications of Pars Plana Vitrectomy for Tractional Retinal Detachment in People With Diabetes: A Systematic Review and Meta-analysis
Source: JAMA Ophthalmol. 2023 Jan 12;141(2):186–95. doi: 10.1001/jamaophthalmol.2022.5817 (PMC9857853; doi:10.1001/jamaophthalmol.2022.5817)
Supplement: Supplement 1. — eFigure 1. Search Structure eFigure 2. PRISMA Flow Diagram eTable 1. Summary of Study Details, Sorted by Study Design eTable 2. Summary of Baseline Patient Characteristics of Included Studies, Sorted by Study Design eTable 3. Summary of Main Outcomes Evaluated in This Review Reported in Eligible RCTs and Prospective Studies (n=8) eTable 4. Summary of Surgical Details of Included Studies, Sorted by Study Design eTable 5. Results of Multivariable Meta-analysis Evaluating the Effect of Baseline Patient Characteristics and Surgical Approaches on Failure to Achieve Retinal Reattachment Following 1 Surgery eTable 6. Frequency of Postoperative Complications [file jamaophthalmol-e225817-s001.pdf]

Supplementary Online Content

McCullough P, Mohite A, Virgili G, Lois N. Outcomes and complication of pars plana vitrectomy for tractional retinal detachment in people with diabetes: a systematic review and meta-analysis. *JAMA Ophthalmol*. Published online January 12, 2023.
doi:10.1001/jamaophthalmol.2022.5817

eFigure 1. Search Structure

eFigure 2. PRISMA Flow Diagram

eTable 1. Summary of Study Details, Sorted by Study Design

eTable 2. Summary of Baseline Patient Characteristics of Included Studies, Sorted by Study Design

eTable 3. Summary of Main Outcomes Evaluated in This Review Reported in Eligible RCTs and Prospective Studies (n=8)

eTable 4. Summary of Surgical Details of Included Studies, Sorted by Study Design

eTable 5. Results of Multivariable Meta-analysis Evaluating the Effect of Baseline Patient Characteristics and Surgical Approaches on Failure to Achieve Retinal Reattachment Following 1 Surgery

eTable 6. Frequency of Postoperative Complications

This supplementary material has been provided by the authors to give readers additional information about their work.

© 2023 McCullough P et al. *JAMA Ophthalmology.*

eFigure 1. Search Structure

MEDLINE	EMBASE
**1** Vitrectomy*<br>**2** Pars Plana Vitrectomy<br>**3** PPV<br>**4** Vitreoretinal surgery<br>**5** VR surgery<br>**6** 1 or 2 or 3 or 4 or 5<br>**7** Diabetes Mellitus*<br>**8** Diabetes Mellitus, Type 1*<br>**9** T1DM<br>**10** Diabetes Mellitus, Type 2*<br>**11** T2DM<br>**12** Insulin dependent diabetes mellitus<br>**13** IDDM<br>**14** Non-insulin dependent diabetes mellitus<br>**15** NIDDM<br>**16** Diabetic retinopathy*<br>**17** DR<br>**18** Proliferative diabetic retinopathy<br>**19** PDR<br>**20** Retinal neovascularization*<br>**21** 7 or 8 or 9 or 10 or 11 or 12 or 13 or 14 or 15 or 16 or 17 or 18 or 19 or 20<br>**22** Tractional retinal detachment<br>**23** TRD<br>**24** 22 or 23<br>**25** 6 and 21 and 24<br>**26** Treatment outcome*<br>**27** Functional<br>**28** Visual<br>**29** Vision, Ocular*<br>**30** Visual acuity*<br>**31** Best corrected visual acuity<br>**32** BCVA<br>**33** Anatomical<br>**34** Retinal reattachment<br>**35** Flat retina<br>**36** Attached retina<br>**37** Success<br>**38** 26 or 27 or 28 or 29 or 30 or 31 or 32 or 33 or 34 or 35 or 36 or 37<br>**39** 25 and 38	**1** Vitrectomy*<br>**2** Pars Plana Vitrectomy*<br>**3** PPV<br>**4** Vitreoretinal surgery*<br>**5** VR surgery<br>**6** 1 or 2 or 3 or 4 or 5<br>**7** Diabetes Mellitus*<br>**8** Diabetes Mellitus, Type 1<br>**9** T1DM<br>**10** Diabetes Mellitus, Type 2<br>**11** T2DM<br>**12** Insulin dependent diabetes mellitus*<br>**13** IDDM<br>**14** Non-insulin dependent diabetes mellitus*<br>**15** NIDDM<br>**16** Diabetic retinopathy*<br>**17** DR<br>**18** Proliferative diabetic retinopathy*<br>**19** PDR<br>**20** Retinal neovascularization*<br>**21** 7 or 8 or 9 or 10 or 11 or 12 or 13 or 14 or 15 or 16 or 17 or 18 or 19 or 20<br>**22** Tractional retinal detachment<br>**23** TRD<br>**24** 22 or 23<br>**25** 6 and 21 and 24<br>**26** Treatment outcome*<br>**27** Functional<br>**28** Visual<br>**29** Vision, Ocular<br>**30** Visual acuity*<br>**31** Best corrected visual acuity<br>**32** BCVA<br>**33** Anatomical<br>**34** Retinal reattachment<br>**35** Flat retina<br>**36** Attached retina<br>**37** Success<br>**38** 26 or 27 or 28 or 29 or 30 or 31 or 32 or 33 or 34 Or 35 or 36 or 37<br>**39** 25 and 38

Search strategy employed for MEDLINE and EMBASE to identify eligible studies evaluating outcomes and complications of pars plana vitrectomy for tractional retinal detachments in people with diabetes mellitus. * indicates that a MeSH term has been used with the search strategy

© 2023 McCullough P et al. *JAMA Ophthalmology.*

eFigure 2. PRISMA Flow Diagram

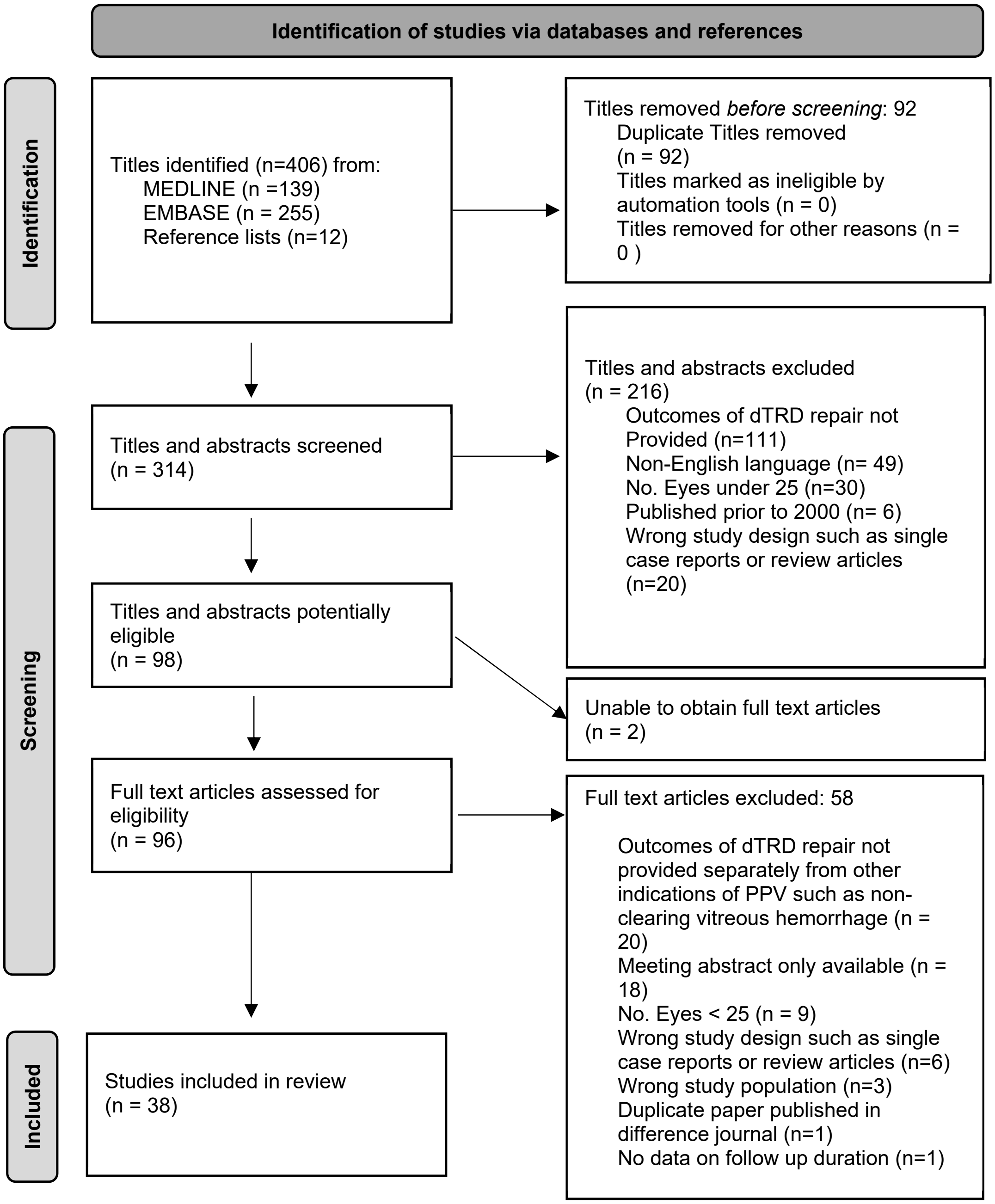

eFigure 2*:* PRISMA flow diagram.

Summary of study inclusion and exclusion process.

dTRD= tractional retinal detachment in people with diabetes mellitus, PPV = pars plana vitrectomy.

© 2023 McCullough P et al. *JAMA Ophthalmology.*

eTable 1. Summary of Study Details, Sorted by Study Design (n = 38)

Study Author and Year	Design	Country of Study	Description of groups within study	Was intra-subject correlation accounted for?	Mean final follow up months (range)	Risk of Bias
Kumar et al 2013	Randomized Controlled Trial^	India	A – used 23G instrumentation<br>B – used 25G instrumentation	NA	24	Fair
Hernandez-Da Mota et al 2010	Randomized Controlled Trial^	Mexico	A – 23G with pre-operative bevacizumab<br>B – 23G without pre-operative bevacizumab	NA	6	Poor
Arevalo et al 2019	Randomized Controlled Trial[+]	The Americas	A – all eyes treated with pre-operative bevacizumab<br>B – no eyes treated with pre-operative bevacizumab	NA	12	Fair
Tao et al 2010	Prospective[b]^	China	NA	No	23 (12-65)	Good
Arevalo et al 2014	Prospective[b]^	Venezuela	NA	NA	24 (12-32)	Fair
Uzel et al 2016	Prospective[b]^	Turkey	A – all eyes treated with pre-operative bevacizumab<br>B – no eyes treated with pre-operative bevacizumab	NA	A – 12.4 (NS)<br>B – 15.1 (NS)	Good
Rashad Qamar et al 2013	Prospective[b]^	Pakistan	NA	No	>12	Good
Elwan et al 2018	Prospective[b]^	Egypt	A – unimanual technique used<br>B – trimanual technique used	No	A – 12 (9-15)<br>B – 11 (9-12)	Fair
Berrocal et al 2018	Retrospective[a]^	Puerto Rico	A – used 27G instrumentation<br>B – used 25G instrumentation	NA	A – 13 (6-34)<br>B – 15 (8-38)	Good
Ricca et al 2020	Retrospective[a]^	USA	NA	No	≥3	Fair
Pokroy et al 2011	Retrospective[a]^	USA	A – pre-operative bevacizumab + age <40 years<br>B - no pre-operative bevacizumab + age <40 years<br>C –pre-operative bevacizumab + age > 40 years<br>D – no pre-opoerative bevacizumab + age > 40 years	No	≥6	Fair
Choovuthayakorn et al 2019	Retrospective[a]^	Thailand	A – all eyes TRD<br>B – all eyes TRD+RRD	NA	≥3	Fair
Parikh et al 2016	Retrospective[a]^	USA	NA	NA	≥6	Fair

© 2023 McCullough P et al. *JAMA Ophthalmology.*

Yang et al 2019	Retrospective[b^]	China	A – all eyes treated with pre-operative 5onbercept<br>B – all eyes treated with pre-operative ranibizumab	No	A – 11.5 (NS)<br>B – 12.2 (NS)	Fair
Rahimy et al 2015	Retrospective[b^]	USA	NA	No	11.1 (NS)	Good
Wang et al 2012	Retrospective[a^]	China	NA	No	9.8 (6-18)	Fair
Dong et al 2016	Retrospective[b^]	China	A – 23 G with pre-operative ranibizumab<br>B – 23G without pre-operative ranibizumab	NA	16 (6-36)	Fair
Wang et al 2016	Retrospective[b^]	China	A – four port + 23G<br>B – three port + 23G	No	A – 12.7 (6-24)<br>B – 16.1 (8-30)	Good
Agarwal et al 2019	Retrospective[b^]	India	NA	NA	3	Fair
Mikhail et al 2017	Retrospective[b^]	Canada	NA	No	30.8 (14.4-59.7)	Fair
Su et al 2014	Retrospective[b^]	Taiwan	NA	NA	29.7 (NS)	Good
Oshima et al 2009	Retrospective[b^]	Japan	NA	No	17.5 (NS)	Good
Fortun et al 2011	Retrospective[b^]	USA	NA	NA	5.7 (NS)	Poor
Sokol et al 2019	Retrospective[b^]	USA	NA	No	11.4 (3-28)	Good
Hu et al 2019	Retrospective[b^]	China	A – no eyes treated with pre-operative bevacizumab<br>B – all eyes treated with pre-operative bevacizumab	Yes	29.1	Good
Gupta et al 2012	Retrospective[a^]	UK	NA	No	14 (3-123)	Fair
Dikopf et al 2015	Retrospective[b^]	USA	NA	No	23.8 (3-78.9)	Good
Larranaga-Fragoso et al 2020	Retrospective[a^]	UK	NA	Yes	≥6	Fair
Gungel et al 2010	Retrospective[b^]	Turkey	A – used 20G instrumentation<br>B – used 23G instrumentation	No	6	Good
Jain et al 2018	Retrospective[b^]	India	NA	NA	≥3	Fair

© 2023 McCullough P et al. *JAMA Ophthalmology.*

Iglicki et al 2019	Retrospective[b+]	Multiple countries	A – all eyes treated with DEX implant<br>B – no eyes treated with DEX implant	NA	≥12	Good
Storey et al 2018	Retrospective[b^]	USA	NA	No	17.5 (NS)	Good
Gupta et al 2012	Retrospective[a+]	UK	NA	Yes	≥12	Fair
La Heij et al 2004	Retrospective[b^]	The Netherlands	NA	Yes	10 (3-36)	Good
Steinmetz et al 2002	Retrospective[b^]	USA	NA	No	16 (6-36)	Good
Algethami et al 2021	Retrospective[b^]	Saudi Arabia	NA	No	21 (NS)	Good
Chen et al 2021	Retrospective[b^]	Taiwan	A – 25G<br>B – 27G	No	≥6	Good
Baek et al 2020	Retrospective[b^]	South Korea	A – silicone oil tamponade without intra-operative bevacizumab<br>B – silicone oil with intra-operative bevacizumab	No	≥12	Good

TRD = tractional retinal detachment, TRD+RRD = combined traction retinal detachment and rhegmatogenous detachment, VH = vitreous hemorrhage, VA = visual acuity, NVI = new vessels on the iris, NS = Not specified, NA = Not Applicable, [a] =patient data was extracted through registries including electronic databases and logbooks, [b] = patient data was extracted directly from clinic records following their review appointment, ^= a single centre study, [+] = a multi-centre study

© 2023 McCullough P et al. *JAMA Ophthalmology.*

eTable 2. Summary of Baseline Patient Characteristics of Included Studies, Sorted by Study Design (n = 38)

Study Author and Year	Design	No. of TRD eyes (patients)	TRD type TRD/TRD+RRD Eyes (%)	Pre-op VH Eyes (%)	Macula attached Eyes (%)	Female Patients (%)	Mean age (range)	Mean Diabetes Duration years (range)	Type 1 Diabetes Mellitus Eyes (Patients)	Mean Baseline HbA1c % (SD)	Systemic Hypertension Eyes (%)	Phakic Eyes (%)	Baseline VA LogMAR (Range)	Pre-op NVI Eyes (%)
Kumar et al 2013	Randomized Controlled Trial	50 (50)	50/0 (100/0)	11 (22)	NS	20 (40)	60.1 (NS)	NS (NS)	NS (NS)	NS (NS)	NS (NS)	26 (52)	1.28 (NS)	0 (0)
Hernandez-Da Mota et al 2010	Randomized Controlled Trial	40 (40)	40/0 (100/0)	0 (0)	40 (0)	16 (40)	55.7 (NS)	NS (NS)	NS (NS)	NS (NS)	22 (55)	NS (NS)	NS (NS)	0 (0)
Arevalo et al 2019	Randomized Controlled Trial	214 (214)	NS	67 (31)	NS	98 (46)	60.4 (NS)	NS (NS)	30 (30)	8.7 (NS)	175 (82)	158 (74)	1.1 (NS)	NS (NS)
Tao et al 2010	Prospective	168 (150)	168/0 (100/0)	49 (29)	99 (59)	73 (49)	52 (20-82)	12 (0.25-36)	NS (21)	NS (NS)	NS (NS)	163 (97)	2.22 (NS)	6 (4)
Arevalo et al 2014	Prospective	114 (114)	114/0 (100/0)	NS	NS	NS (NS)	45 (21-85)	NS (NS)	NS (NS)	NS (NS)	NS (NS)	NS (NS)	NS (NS)	NS (NS)
Uzel et al 2016	Prospective	78 (78)	78/0 (100/0)	17 (22)	0 (0)	44 (56)	56.2 (NS)	14.3 (NS)	NS (NS)	NS (NS)	25 (32)	19 (24)	1.80 (NS)	3 (4)
Rashad Qamar et al 2013	Prospective	84 (75)	84/0 (100/0)	36 (43)	49 (58)	40 (53)	52 (40-60)	11.4 (10-36)	NS (11)	NS (NS)	NS (NS)	79 (94)	2 (NS)	NS (NS)
Elwan et al 2018	Prospective	82 (67)	68/14 (81/19)	40 (49)	NS	30 (45)	54.5 (44-63)	15.4 (11-25)	NS (20)	NS (NS)	NS (NS)	33 (40)	1.79 (NS)	NS (NS)
Berrocal et al 2018	Retrospective	42 (42)	42/0 (100/0)	0 (0)	0 (0)	19 (45)	52.0 (33-72)	23.0 (8-35)	NS (NS)	8.3 (NS)	NS (NS)	NS (NS)	0.85 (0.40-1.85)	NS (NS)
Ricca et al 2020	Retrospective	240 (203)	212/28 (88/12)	0 (0)	NS	97 (48)	48.0 (21-88)	NS (NS)	NS (NS)	NS (NS)	NS (NS)	215 (90)	1.2 (NS)	NS (NS)
Pokroy et al 2011	Retrospective	99 (90)	89/10 (90/10)	71 (72)	0 (0)	44 (49)	51.0 (26-76)	17.5 ((NS)	40 (40)	9.0 (NS)	90 (91)	82 (83)	1.39 (0-2.3)	6 (6)
Choovuthayakorn et al 2019	Retrospective	455 (455)	326/129 (72/28)	0 (0)	NS	298 (65)	48.8 (NS)	NS (NS)	27 (5.9)	NS (NS)	NS (NS)	NS (NS)	1.6 (NS)	NS (NS)
Parikh et al 2016	Retrospective	40 (37)	16/24 (40/60)	23 (58)	7 (17.5)	14 (38)	46.9 (23-68)	25.4 (4-31)	NS (11)	NS (NS)	34 (85)	35 (88)	NS (NS)	NS (NS)
Yang et al 2019	Retrospective	79 (74)	70/9 (88/12)	34 (43)	20 (25)	34 (46)	50.6 (28-73)	NS (NS)	NS (NS)	7.7 (NS)	57 (72)	67 (85)	1.27 (0.50-2.30)	8 (10)
Rahimy et al 2015	Retrospective	62 (58)	53/9 (85/15)	44 (71)	9 (14.5)	23 (40)	48.1 (22-63)	NS (NS)	NS (NS)	8.4 (1.9)	NS (NS)	57 (92)	2 (NS)	NS (NS)

© 2023 McCullough P et al. *JAMA Ophthalmology.*

Wang et al 2012	Retrospective	30 (24)	25/5 (83/17)	25 (83)	0 (0)	15 (63)	57.1 (37-82)	11.4 (3-20)	NS (NS)	NS (NS)	NS (NS)	NS (NS)	NS (NS)	NS (NS)
Dong et al 2016	Retrospective	97 (97)	97/0 (100/0)	0 (0)	0 (0)	NS (NS)	52 (24-79)	12 (1-30)	NS (NS)	NS (NS)	NS (NS)	NS (NS)	1.91 (NS)	0 (0)
Wang et al 2016	Retrospective	66 (58)	46/20 (70/30)	21 (32)	24 (36)	36 (62)	52.2 (25-75)	NS (NS)	NS (27)	NS (NS)	NS (NS)	60 (91)	2.12 (0.78-2.3)	5 (8)
Agarwal et al 2019	Retrospective	34 (34)	34/0 (100/0)	0 (0)	NS	NS (NS)	55.6 (NS)	NS (NS)	NS (NS)	NS (NS)	NS (NS)	NS (NS)	NS (NS)	NS (NS)
Mikhail et al 2017	Retrospective	109 (73)	61/48 (56/44)	35 (32)	46 (42)	34 (47)	53.9 (NS)	17.4 (1-44)	NS (41)	NS (NS)	NS (NS)	76 (70)	1.17 (NS)	NS (NS)
Su et al 2014	Retrospective	32 (32)	32/0 (100/0)	0 (0)	0 (0)	18 (56)	48.9 (NS)	NS (NS)	NS (NS)	NS (NS)	NS (NS)	31 (97)	1.56 (NS)	NS (NS)
Oshima et al 2009	Retrospective	33 (26)	28/5 (85/15)	12 (36)	20 (61)	11 (42)	54.5 (28-67)	NS (NS)	NS (NS)	7.3 (1.1)	NS (NS)	29 (88)	1.13 (1.1-2.3)	6 (18)
Fortun et al 2011	Retrospective	30 (28)	NS	NS	NS	NS (NS)	NS (NS)	NS (NS)	NS (NS)	NS (NS)	NS (NS)	NS (NS)	NS (NS)	NS (NS)
Sokol et al 2019	Retrospective	69 (61)	38/31 (55/45)	0 (0)	21 (30)	25 (41)	47 (21-65)	NS (NS)	NS (NS)	9 (NS)	NS (NS)	56 (81)	1.84 (NS)	NS (NS)
Hu et al 2019	Retrospective	136 (NS)	136/0 (100/0)	100 (74)	NS	NS (NS)	NS (NS)	NS (NS)	NS (NS)	NS (NS)	NS (NS)	NS (NS)	NS (NS)	NS (NS)
Gupta et al 2012	Retrospective	181 (NS)	NS	NS	NS	NS (NS)	NS (NS)	NS (NS)	62 (NS)	NS (NS)	NS (NS)	NS (NS)	1.61 (NS)	NS (NS)
Dikopf et al 2015	Retrospective	70 (55)	21/49 (30/70)	53 (76)	31 (44)	32 (58)	47.7 (23-76)	NS (NS)	NS (NS)	NS (NS)	NS (NS)	66 (94)	1.59 (NS)	NS (NS)
Larranaga-Fragoso et al 2020	Retrospective	109 (109)	109/0 (100/0)	0 (0)	NS	61 (56)	52.2 (23-75)	NS (NS)	30 (30)	8.9 (NS)	55 (50)	NS (NS)	1.33 (0.3-3)	NS (NS)
Gungel et al 2010	Retrospective	49 (43)	43/6 (88/12)	23 (47)	43 (88)	19 (44)	52.8 (NS)	NS (NS)	NS (NS)	NS (NS)	NS (NS)	NS (NS)	2.32 (0.52-3.1)	NS (NS)
Jain et al 2018	Retrospective	33 (33)	23/10 (70/30)	0 (0)	0 (0)	16 (48)	NS (NS)	NS (NS)	NS (NS)	NS (NS)	NS (NS)	NS (NS)	1.8 (NS)	NS (NS)
Iglicki et al 2019	Retrospective	148 (148)	148/0 (100/0)	0 (0)	0 (0)	64 (43)	61.8 (NS)	NS (NS)	NS (NS)	8.0 (NS)	NS (NS)	142 (96)	0.97 (NS)	NS (NS)

© 2023 McCullough P et al. *JAMA Ophthalmology.*

Storey et al 2018	Retrospective	403 (359)	317/86 (79/21)	322 (80)	161 (40)	153 (43)	49.6 (26-69)	NS (NS)	NS (NS)	8.4 (NS)	NS (NS)	NS (NS)	1.73 (0.3-2.28	NS (NS)
Gupta et al 2012	Retrospective	117 (NS)	117/0 (100/0)	NS	NS	NS (NS)	NS (NS)	NS (NS)	NS (NS)	NS (NS)	NS (NS)	NS (NS)	NS (NS)	NS (NS)
La Heij et al 2004	Retrospective	44 (33)	44/0 (100/0)	35/9 (80/20)	NS	19 (56)	NS (NS)	NS (NS)	NS (NS)	NS (NS)	NS (NS)	41 (93)	1.6 (0.4-2.7)	NS (NS)
Steinmetz et al 2002	Retrospective	67 (62)	67/0 (100/0)	52 (78)	37 (40)	32 (52)	46 (25-74)	NS (NS)	NS (NS)	NS (NS)	NS (NS)	63 (94)	NS (NS)	0 (0)
Algethami et al 2021	Retrospective	46 (43)	36/10 (78/22)	NS	15 (33)	15 (35)	51.5 (NS)	NS (NS)	10 (23)	NS (NS)	NS (NS)	NS (NS)	1.62 (0.18-3.00)	NS (NS)
Chen et al 2021	Retrospective	43 (42)	100/0 (100/0)	NS (NS)	NS (NS)	21 (50)	53.1 (29-68)	8.97 (0.5-20)	NS (NS)	8.60 (5.7-14.2)	NS (NS)	NS (NS)	1.58 (0.05-2.8)	NS (NS)
Baek et al 2020	Retrospective	46 (44)	46/0 (100/0)	0 (0)	0 (0)	20 (45)	54.7 (NS)	6.8 (NS)	NS (NS)	8.8 (NS)	12 (26)	NS (NS)	1.20 (NS)	NS (NS)

TRD = tractional retinal detachment, TRD+RRD = combined traction retinal detachment and rhegmatogenous detachment, VH = vitreous hemorrhage, VA = visual acuity, NVI = new vessels on the iris, NS = Not specified

© 2023 McCullough P et al. *JAMA Ophthalmology.*

eTable 3. Summary of Main Outcomes Evaluated in This Review Reported in Eligible RCTs and Prospective Studies (n=8)

**Study Author and Year**	Design	Description of groups within study	**Visual Acuity at final follow up LogMAR ± SD**	**Retinal re-deattachement after one attempt Eyes (%)**	**Retinal re-deattachment after more than one attempt Eyes (%)**
Kumar et al 2013	Randomized Controlled Trial	A – used 23G instrumentation<br>B – used 25G instrumentation	A: 1.03 ± 0.41<br>B: 0.88 ± 0.45	-	-
Hernandez-Da Mota et al 2010	Randomized Controlled Trial	A – 23G with pre-operative bevacizumab<br>B – 23G without pre-operative bevacizumab	A: 0.76 ± 0.14<br>B: 2.07 ± 0.18	-	-
Arevalo et al 2019	Randomized Controlled Trial	A – all eyes treated with pre-operative bevacizumab<br>B – no eyes treated with pre-operative bevacizumab	A: 0.60 ± 0.48<br>B: 0.7 ± 0.31	A: 6 (5.8)<br>B: 14 (12.5)	A: 0 (0)<br>B: 0 (0)
Tao et al 2010	Prospective	NA	1.24 ± 1.00	10 (6.3)	10 (6.3)
Arevalo et al 2014	Prospective	NA	-	0 (0)	-
Uzel et al 2016	Prospective	A – all eyes treated with pre-operative bevacizumab<br>B – no eyes treated with pre-operative bevacizumab	A: 1.52 ± 0.29<br>B: 1.78 ± 0.33	A: 2 (5.3)<br>B: 3 (7.5)	-
Rashad Qamar et al 2013	Prospective	NA	1.24 ± 1.22	6 (7.1)	-
Elwan et al 2018	Prospective	A – unimanual technique used<br>B – trimanual technique used	A: 1.25 ± 0.2<br>B: 1.18 ± 0.16	A: 4 (8.1)<br>B: 2 (5.3)	A: 0 (0)<br>B: 0 (0)

A and B denote study arms with different intervenions within a study.

© 2023 McCullough P et al. *JAMA Ophthalmology.*

eTable 4. Summary of Surgical Details of Included Studies, Sorted by Study Design (n = 38)

**Study Author and Year**	Design	**No. of TRD eyes (patients)**	**PPV/PPV+Phaco/PPV+lensectomy/PPV+Scleral buckle Eyes (Percentage)**	**Surgical technique**	**Gauge 20G/23G/25G/27G Eyes (Percentage)**	**Tamponade None or BSS or Air/SF6 or C3F8/ Silicone oil/ PFO Eyes (Percentage)**	**Pre-op or Intra-op Anti-VEGF agent**	**Pre-op or intra-op AntiVEGF Eyes (Percentage)**
Kumar et al 2013	Randomized Controlled Trial	50 (50)	50/0/0/0 (100/0/0/0)	Combination of segmentation and delamination	0/25/25/0 (0/50/50/0)	0/43/7/0 (0/86/14/0)	None	NA (NA)
Hernandez-Da Mota et al 2010	Randomized Controlled Trial	40 (40)	40/0/0/0 (100/0/0/0)	Combination of segmentation , peeling and en-bloc dissection	0/40/0/0 (0/100/0/0)	NS (NS)	Bevacizumab	20 (50)
Arevalo et al 2019	Randomized Controlled Trial	214 (214)	214/0/0/0 (100/0/0/0)	NS	0/174/27/13 (0/81/13/6)	NS/NS/72/NS (NS/NS/34/NS)	Bevacizumab	102 (48)
Tao et al 2010	Prospective	168 (150)	135/33/0/0 (80/20/0/0)	NS (NS)	168/0/0/0 (100/0/0/0)	168/0/0/0 (100/0/0/0)	None	NA (NA)
Arevalo et al 2014	Prospective	114 (114)	114/0/0/0 (100/0/0/0)	Proportional reflux hydrodissection	0/114/0/0 (0/100/0/0)	NS/NS/0/NS (NS/NS/0/NS)	Bevacizumab	114 (100)
Uzel et al 2016	Prospective	78 (78)	53/25/0/0 (68/32/0/0)	Combination of segmentation and delamination	0/78/0/0 (0/100/0/0)	20/20/38/0 (26/26/48/0	Bevacizumab	38 (49)
Rashad Qamar et al 2013	Prospective	84 (75)	51/33/0/0 (61/39/0/0)	Bimanual segmentation	84/0/0/0 (100/0/0/0)	84/0/0/0 (100/0/0/0)	None	NA (NA)
Elwan et al 2018	Prospective	82 (67)	59/23/0/0 (72/28/0/0)	Unimanual delamination and removal; trimanual delamination	0/82/0/0 (0/100/0/0)	0/0/44/0 (0/0/100/0)	None	NA (NA)
Berrocal et al 2018	Retrospective	42 (42)	42/0/0/0 (100/0/0/0)	All probe lift and shave	0/0/22/20/ (0/0/52/48)	41/1/0/0 (98/2/0/0)	NS	NS (NS)
Ricca et al 2020	Retrospective	240 (203)	NS (NS)	NS	NS (NS)	NS (NS)	NS	NS (NS)

© 2023 McCullough P et al. *JAMA Ophthalmology.*

Pokroy et al 2011	Retrospective	99 (90)	98/0/1/0<br>(99/0/1/0)	Combination of segmentation and delamination	99/0/0/0<br>(100/0/0/0)	33/62/4/0<br>(33/63/4/0)	Bevacizumab	34 (34)
Choovuthayakorn et al 2019	Retrospective	455 (455)	365/90/0/0<br>(80/20/0/0)	NS	246/209/0/0<br>(54/46/0/0)	NS/NS/184/NS<br>(NS/NS/40/NS)	Bevacizumab	239 (53)
Parikh et al 2016	Retrospective	40 (37)	31/0/9/0<br>(78/22)	En-bloc dissection	2/38/0/0<br>(5/95/0/0)	0/0/40/0<br>(0/0/100/0)	None	NA (NA)
Yang et al 2019	Retrospective	79 (74)	79/0/0/0<br>(100/0/0/0)	Combination of segmentation and delamination	NS (NS)	0/0/79/0<br>(0/0/100/0)	Conbercept<br>Ranibizumab	37 (47)<br>42 (53)
Rahimy et al 2015	Retrospective	62 (58)	41/21/0/0<br>(67/33/0/0)	NS	NS (NS)<br>Mix of 20G and 23G	23/23/16/0<br>(37/37/26/0)	Bevacizumab	8 (13)
Wang et al 2012	Retrospective	30 (24)	14/NS/NS/0<br>(47/NS/NS/0)*	Combination of segmentation and delamination	30/0/0/0<br>(100/0/0/0)	0/15/15/0<br>(0/50/50/0)	None	NA (NA)
Dong et al 2016	Retrospective	97 (97)	97/0/0/0<br>(100/0/0/0)	NS	0/97/0/0<br>(0/100/0/0)	NS/15/59/NS<br>(NS/15/61/NS)	Ranibizumab	47 (48)
Wang et al 2016	Retrospective	66 (58)	11/55/0/0<br>(17/83/0/0)	Bimanual segmentation; Trimanual segmentation	0/66/0/0<br>(0/100/0/0)	0/36/30/0<br>(0/55/45/0)	None	NA (NA)
Agarwal et al 2019	Retrospective	34 (34)	34/0/0/0<br>(100/0/0/0)	Proportional reflux hydrodissection	0/0/34/0<br>(0/0/100/0)	NS (NS)<br>Mix of C3F8 and silicone oil	Ranibizumab	34 (100)
Mikhail et al 2017	Retrospective	109 (73)	81/28/0/0<br>(74/26/0/0)	Bimanual segmentation	0/0/109/0<br>(0/0/100/0)	41/60/8/0<br>(38/55/7/0)	None	NA (NA)
Su et al 2014	Retrospective	32 (32)	32/0/0/0<br>(100/0/0/0)	Combination of segmentation and delamination	32/0/0/0<br>(100/0/0/0)	0/32/0/0<br>(0/100/0/0)	Bevacizumab	32 (100)
Oshima et al 2009	Retrospective	33 (26)	11/22/0/0<br>(33/67/0/0)	Combination of segmentation and delamination	33/0/0/0<br>(100/0/0/0)	NS/NS/1/0<br>(NS/NS/3/0)<br>Mixed use of air and long-acting gas but NS	None	NA (NA)
Fortun et al 2011	Retrospective	30 (28)	30/0/0/0<br>(100/0/0/0)	Viscodissection	0/0/30/0<br>(0/0/100/0)	NS/NS/0/NS<br>(NS/NS/0/NS)	NS	NS (NS)
Sokol et al 2019	Retrospective	69 (61)	52/6/3/8<br>(75/9/4/12)	Delamination and removal of membranes	0/69/0/0<br>(0/100/0/0)	0/63/6/0<br>(0/91/9/0)	None	NA (NA)
Hu et al 2019	Retrospective	136 (NS)	NS**	NS	0/136/0/0<br>(0/100/0/0)	31/40/65/0<br>(23/29/48/0)	Bevacizumab	70 (51)

© 2023 McCullough P et al. *JAMA Ophthalmology.*

Gupta et al 2012	Retrospective	181 (NS)	NS**	En-bloc dissection	181/0/0/0<br>(100/0/0/0)	73/95/13/0<br>(40/52/7/0)	NS	NS (NS)
Dikopf et al 2015	Retrospective	70 (55)	60/NS/NS/0<br>(86/NS/NS/0)^	NS (NS)	0/0/70/0<br>(0/0/100/0)	21/44/5/0<br>(30/63/7/0)	None	NA (NA)
Larranaga-Fragoso et al 2020	Retrospective	109 (109)	NS (NS)	Combination of segmentation and delamination	0/109/0/0<br>(0/100/0/0)	58/24/27/0<br>(53/22/25/0)	NS	7 (6)
Gungel et al 2010	Retrospective	49 (43)	0/49/0/0<br>(0/100/0/0	NS	23/26/0/0<br>(47/53/0/0)	0/7/42/0<br>(0/14/86/0)	Bevacizumab	49 (100)
Jain et al 2018	Retrospective	33 (33)	33/0/0/0<br>(100/0/0/0)	Proportional reflux hydrodissection	0/0/33/0<br>(0/0/100/0)	20/3/4/6<br>(61/9/12/18)	Ranibizumab	15 (45)
Iglicki et al 2019	Retrospective	148 (148)	131/17/0/0<br>(89/11/0/0)	NS	NS (NS)<br>Mixed use of 23G and 25G	0/0/148/0<br>(0/0/100/0)	None	NA (NA)
Storey et al 2018	Retrospective	403 (359)	383/0/13/7<br>(95/0/3/2)	NS	40/114/247/2<br>(10/28/61/1)	178/127/98/0<br>(44/32/24/0)	Bevacizumab	23 (6)
Gupta et al 2012	Retrospective	117 (NS)	NS (NS)	NS	117/0/0/0<br>(100/0/0/0)	NS (NS)	Bevacizumab	30 (26)
La Heij et al 2004	Retrospective	44 (33)	38/0/6/0<br>(86/0/14/0)	Bimanual segmentation	44/0/0/0<br>(100/0/0/0)	25/9/10/0<br>(57/21/23/0)	None	NA (NA)
Steinmetz et al 2002	Retrospective	67 (62)	37/0/4/16<br>(55/0/6/24)	NS	67/0/0/0<br>(100/0/0/0)	27/39/1/0<br>(40/58/2/0)	None	NA (NA)
Algethami et al 2021	Retrospective	46 (43)	46/0/0/0<br>(100/0/0)	NS	NS (NS)	NS/10/15/NS<br>(NS/21/33/NS)	NS	NS (NS)

© 2023 McCullough P et al. *JAMA Ophthalmology.*

Chen et al 2021	Retrospective	43 (42)	43/0/0/0<br>(100/0/0/0)	NS	0/0/22/21<br>(0/0/51/49)	16/19/8/0<br>(37/44/19/0)	Bevacizumab<br>Ranibizumab	37 (86)
Baek et al 2020	Retrospective	46 (44)	14/32/0/0<br>(30/70/0/0)	Combination of segmentation and delamination	0/0/46/0<br>(0/0/100/0)	0/0/46/0<br>(0/0/100/0)	Bevacizumab	20 (43)

TRD = tractional retinal detachments; PPV= pars plana vitrectomy only; PPV+Phaco = pars plana vitrectomy with phacoemulsification and IOL implantation; PPV + lensectomy = pars plana vitrectomy with lensectomy and IOL implantation;; PPV+ Scleral buckle = pars plana vitrectomy with scleral buckle, None or BSS or Air = no post-operative tamponade or balanced salt solution or air post-operative tamponade; SF6 or C3F8= SF6 or C3F8 gas post-operative tamponade; PFO = perfluoro-n-octane liquid tamponade, NS = not specified

*Cataract surgery (type of surgery not specified) occurred in 16 eyes alongside PPV.

**Mixed PPV only and PPV+Phacoemulsification and intraocular lens implantation; proportions not specified

^ Cataract surgery (type of surgery not specified) occurred in 10 eyes alongside PPV.

© 2023 McCullough P et al. *JAMA Ophthalmology.*

eTable 5. Results of Multivariable Meta-analysis Evaluating the Effect of Baseline Patient Characteristics and Surgical Approaches on Failure to Achieve Retinal Reattachment Following 1 Surgery

Random-effects meta-regression method: Restricted Maximum likelihood (REML)					Number of obs = 28<br>Method: REML<br>Residual heterogeneity:<br>tau2 = 4.8e-07<br>I2 (%) = 0.00<br>H2 = 1.00<br>R-squared (%) = 33.61<br>Wald chi2(5) = 0.03<br>Prob > chi2 = 1.0000	
**Covariate**	**Coefficient**	**Standard error**	**Z**	**P> |z|**	**[95% interval]**	
VH	0.2359131	17.6812	0.01	0.989	-34.41859	34.89042
Anti VEGF	1.005576	13.38035	0.08	0.940	-25.21943	27.23058
23-27G	0.8378869	18.90082	0.04	0.965	-36.20704	37.88282
Phakic	0.0345505	0.3960991	0.09	0.930	-.0741789	0.8108904
**Combined**	**-1.569015**	**38.83542**	**-0.04**	**0.968**	**-77.68505**	**74.54702**
**Tests of residual homogeneity:** Q_res = chi2(22) = 0.20 Prob > Q_res = 1.0000						

VH = vitreous hemorrhage, anti-VEGF = anti-vascular endothelial growth factor drugs, 23-25G = includes 23g, 25g and 27g small gauge vitrectomy, phakic = eyes which have their physiological crystalline lens. REML = Restricted maximum likelihood.

© 2023 McCullough P et al. *JAMA Ophthalmology.*

eTable 6. Frequency of Postoperative Complications

A. Relating to the Retina and the Choroid

Post-operative complication	No. of studies reporting post-operative complication	Total No. eyes within studies	Mean post-operative complication rate percentage, % (range)
Choroidal detachment	3	182	0 (0.0-0.0)
Choroidal hemorrhage	2	290	0 (0.0-0.0)
ERM	7	500	7.9 (4.0-24.3)
Fibrin formation	4	247	22.3 (1.4-45.5)
Pre-retinal bleeding	1	50	2.00 (NA)
Progressive retinal fibrosis	7	470	2.9 (0.0-9.1)
PVR	1	148	8.8 (NA)
VH	22	1889	22.5 (0.0-65.0)

Abbreviations: ERM, epiretinal membrane; NS, not specified; PVR, proliferative vitreoretinopathy; TRD, tractional retinal detachment; VH, vitreous hemorrhage.

B. Relating to Tamponade Agent Used

Post-operative complication	No. of studies reporting post-operative complication	Total No. eyes within studies	Mean post-operative complication rate, % (range)
Oil emulsification	1	40	7.5 (NA)
Oil migration to anterior chamber	1	40	10.0 (NA)
Oil migration under the conjunctiva	1	40	5.0 (NA)
Subretinal oil	1	40	5.0 (NA)

Abbreviations: NA, Not applicable as not eyes had a silicone oil tamponade; NS, not specified; TRD, tractional retinal detachment.

C. Relating to Intraocular Pressure

Post-operative complication	No. of studies reporting post-operative complication	Total No. eyes within studies	Mean post-operative complication rate, % (range)
Glaucoma	2	70	4.2 (3.3-5.0)
Hypotony	7	449	3.9 (0.0-10.6)
IOP increase	9	883	10.5 (0.0-35.7)

Abbreviations: IOP increase, intraocular pressure increase; NS, not specified; TRD, tractional retinal detachment.

© 2023 McCullough P et al. *JAMA Ophthalmology.*

D. Relating to New Vessel Development or Progression

Post-operative complication	No. of studies reporting post-operative complication	Total No. eyes within studies	Mean post-operative complication rate, % (range)
Hyphemia	2	73	2.75 (2.5-3.0)
NVG	14	1445	5.3 (1.4-18.2)
NVI	4	354	4.8 (1.0-8.2)

Abbreviations: NVG, neovascular glaucoma; NVI, new vessels on the iris; NS, not specified; TRD, tractional retinal detachment;

E. Miscellaneous Postoperative Complications

Post-operative complication	No. of studies reporting post-operative complication	Total No. eyes within studies	Mean post-operative complication rate, % (range)
Anterior chamber exudate formation	1	82	6.1 (NA)
Cataract formed during follow up period	5	356	20.0 (6-28)
Corneal edema	1	40	10.0 (NA)
Endophthalmitis	7	857	0.0 (0-0)
Phthisis bulbi	2	192	1.09 (0.9-1.3)

Abbreviations: NA, Not applicable as all PPV with cataract surgery; NS, not specified; PPV, pars plana vitrectomy; TRD, tractional retinal detachment.

© 2023 McCullough P et al. *JAMA Ophthalmology.*
